# **SAPID**: A **S**trategy to **A**nalyze **P**lant Extracts Taste **I**n **D**epth. Application to the complex taste of *Swertia chirayita* (Roxb.) H.Karst.

**DOI:** 10.1016/j.crfs.2025.101043

**Published:** 2025-04-05

**Authors:** Adriano Rutz, Pascale Deneulin, Ivano Tonutti, Benoît Bach, Jean-Luc Wolfender

**Affiliations:** a Institute of Molecular Systems Biology, ETH Zürich, Otto-Stern-Weg 3, Zürich, 8049, Switzerland; b School of Pharmaceutical Sciences, University of Geneva, Rue Michel-Servet 1, Geneva, 1205, Switzerland; c Institute of Pharmaceutical Sciences of Western Switzerland, University of Geneva, Rue Michel-Servet 1, Geneva, 1205, Switzerland; d Changins – Viticulture and Oenology, University of Applied Sciences and Arts Western Switzerland, Rte de Duillier 50, Nyon, 1260, Switzerland; e TRADALL S.A. (Bacardi Group), Rte de Meyrin 265, Meyrin, 1217, Switzerland

**Keywords:** Mass spectrometry, Metabolomics, Natural extracts, Taste

## Abstract

Analyzing bitterness is challenging because of the diverse range of bitter compounds, the variability in sensory perception, and its complex interaction with other tastes. To address this, we developed an untargeted approach to deconvolute the taste and molecular composition of complex plant extracts. We applied our methodology to an ethanolic extract of *Swertia chirayita* (Roxb.) H.Karst., a plant recognized for its distinctive bitterness. Chemical characterization was performed through nuclear magnetic resonance spectroscopy experiments together with untargeted liquid chromatography-high resolution tandem mass spectrometry analysis coupled to a charged aerosol detector. After clustering the fractions based on chemical similarity, we performed free sensory analysis and classical descriptive analysis on each cluster. Our results confirmed the attribution of bitterness to iridoids and highlighted the role of other important compounds in the overall taste. This method provides a systematic approach for analyzing and potentially enhancing the taste profiles of plant-based beverages.

## Highlights

• An untargeted method analyzes plant extracts’ taste and chemical composition.
• Bitterness of *Swertia chirayita* extract linked to major and minor iridoids.
• *Chemically informed tasting* reveals subtle tastes adding to extract complexity.
• Insights gained on sub-threshold taste impacts and taste modulation properties.

## Graphical abstract

**Figure:**
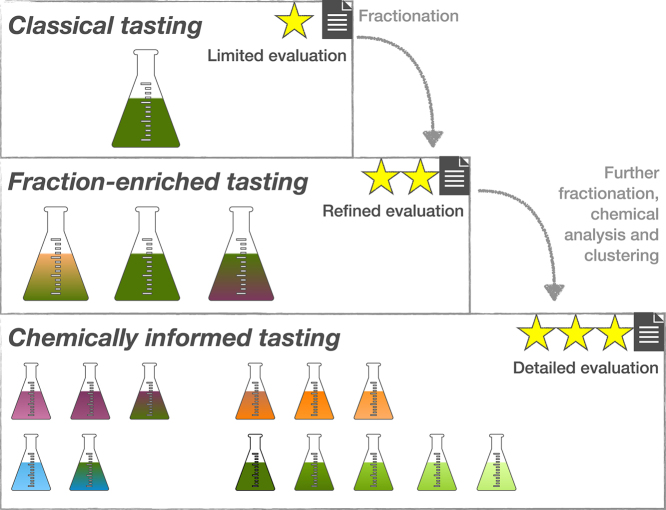

## 1 Introduction

The exploration of bitter compounds in beverages represents a rapidly evolving area of research with significant implications for both the food and beverage industries and human health (Joshi et al., 2006). Bitter compounds, naturally present in ingredients such as citrus peels, hops, and various botanicals, contribute to the complex flavor profiles increasingly sought after in products ranging from craft beers to functional drinks (Yan and Tong, 2022). Historically, bitterness was often minimized in formulations due to consumer aversion, but recent trends reflect a growing acceptance and even preference for bitter flavors among niche markets and health-conscious demographics (Belitz and Wieser, 1985, Zhao et al., 2022). This shift is partly driven by the recognition that certain bitter compounds may offer health benefits (Barratt-Fornell and Drewnowski, 2002) while excessive bitterness, if unbalanced, can lead to product rejection (Gaudette and Pickering, 2013). Thus, understanding the sources, perception, and modulation of bitterness is critical for optimizing sensory appeal alongside harnessing the functional benefits of bioactive compounds. Conventional approaches have predominantly focused on isolating and quantifying single taste-active compounds, a strategy that often fails to capture the full complexity of natural taste profiles. In contrast, the work presented here introduces an innovative, holistic approach that leverages high-end mass spectrometry and advanced data analysis to deconvolute complex taste profiles, thereby transcending classical methodologies that highlight individual taste components. Our *chemically informed tasting* strategy integrates untargeted mass spectrometric analysis with automated composition assessment and sophisticated sensory evaluation, providing a comprehensive view that encompasses both known and novel taste-active constituents.

*Swertia chirayita* (Roxb.) H.Karst., a bitter plant of significant medicinal importance in India, exemplifies both the challenges and opportunities inherent in this holistic approach. Growing between 1200 and 3200 m in the Himalayan range (Shukla et al., 2014), *S. chirayita* is renowned for its medicinal properties (Saha and Das, 2010, Phoboo et al., 2012, Suryawanshi et al., 2006) and is currently a critically endangered species (Kumar and Van Staden, 2016). As with other Gentianaceae, it is known to contain high levels of xanthones and iridoids (Hostettmann-Kaldas et al., 1981, Sharma et al., 2022), with Amarogentin standing out as one of the most bitter compounds known, exhibiting a bitter value of 58,000,000 (Wölfle and Schempp, 2018). The inherent chemical and sensory diversity of *S. chirayita* extracts poses significant analytical challenges that demand advanced methodologies for accurate deconvolution at a molecular level. To address this, we further investigated an enriched ethanolic extract of *S. chirayita* previously analyzed in Rutz and Wolfender (2023) using our *chemically informed tasting* approach. Our workflow combines fraction enrichment with quantitative chemical evaluation via Nuclear Magnetic Resonance (NMR) spectroscopy and automated composition assessment through high resolution tandem mass spectrometry (UHPLC-HRMS/MS) coupled with photo diode array detection (PDA-DAD) and charged aerosol detection (CAD). Parallel sensory evaluation was performed using methods such as napping (Pagès, 2003) and free descriptive profiling, with intensity assessments further substantiating the sensory relevance of our chemical findings. This untargeted, integrative methodology not only facilitates the deconvolution of complex taste profiles but also embodies a paradigm shift towards a holistic vision in taste research, paving the way for more informed and effective food and beverage formulations.

**Fig. 1:**
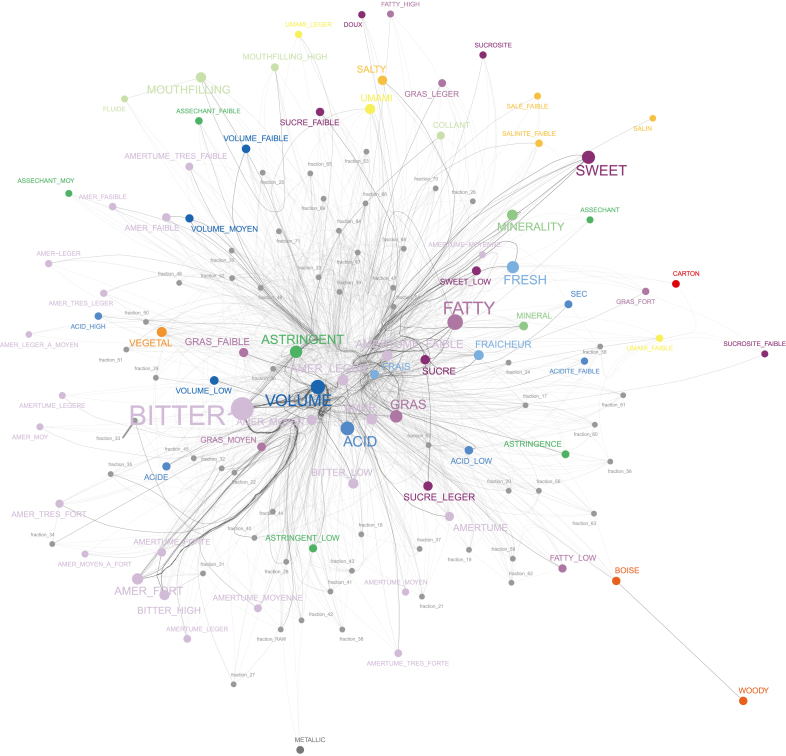
**Network of the Generated Taste Descriptors.** Descriptors assigned to the same fraction are linked together. The color of each edge represents the assigned category, and the font size and edge thickness reflect the frequency of occurrence. As anticipated, the ‘Bitter’ category is the most represented.

## 2 Results and discussion

### 2.1 Fractionation and clustering

The initial step in unraveling the intricate taste profile of *S. chirayita* was the comprehensive fractionation of its extract. The raw extract was first freeze-dried and then subjected to Vacuum Liquid Chromatography (VLC), as detailed in Section 4.3.1. The ethanolic VLC fraction was subsequently further fractionated following the procedure in Section 4.3.2, yielding a total of 82 fractions. A recovery rate of 78% of the initial 10 g mass was achieved, as summarized in the table provided in Appendix A of Supplementary data. Out of these 82 fractions, 53 (specifically, fractions 17 to 71) were selected for further analysis because earlier fractions were empty due to the VLC enrichment, fraction 49 was empty, and fractions above 71 were too apolar. This fractionation process was critical for both concentrating the molecular constituents and effectively separating them to highlight subtle taste differences. The separation enabled structured tasting sessions, where similar fractions could be evaluated collectively. Subsequently, the fractions were analyzed by HRMS/MS and NMR to obtain chemical insights that would underpin our *chemically informed tasting* approach. The similarity among fractions was assessed via their ^1^H NMR signals, providing a generic quantitative view of the major compounds present. Detailed clustering of the NMR signals is presented in Appendix B of Supplementary data. Finally, the raw extract and derived fractions were characterized by UHPLC-HRMS/MS coupled with Photo Diode Array (PDA) detection and Charged Aerosol Detection (CAD). Comparative chromatograms of the enriched extract and selected fractions are available in Appendix C of Supplementary data.

**Fig. 2:**
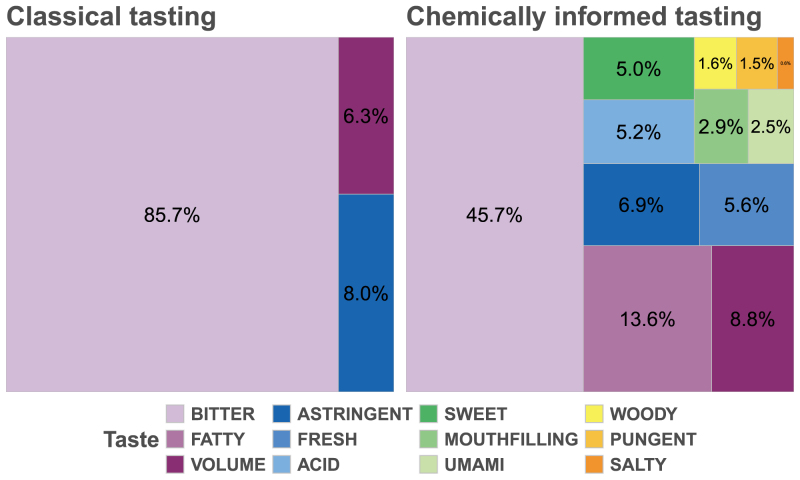
**Comparison of Classical and *Chemically Informed Tasting*.** The summed intensity scores (reported by at least two panelists) normalized by the mass of each fraction are displayed. For the crude extract (left), only three taste attributes were detected, with bitterness dominating at over 85% of the overall intensity. In contrast, the *chemically informed tasting* approach (right) uncovered nine additional taste attributes, thereby revealing a richer and more nuanced sensory profile.

### 2.2 Tasting

To avoid sensory saturation, each session was limited to a maximum of seven fractions per panelist. At the beginning of each session, panelists were provided, under blind conditions, with a glass of Chasselas (a non-aromatic white wine) that served as a palate cleanser and a consistent standard. Panelists were asked to score its intensity using the nine sensory descriptors on which they had been trained (a summary of score variations is available in Appendix D of Supplementary data). Subsequently, a group of fractions was evaluated using the ‘napping’ method (Pagès, 2005). In this method, panelists arranged the tasting glasses on a rectangular sheet of paper based on their perceived global similarities or dissimilarities, placing glasses closer together if the tastes were deemed similar. This approach allowed the capture of holistic sensory impressions that might not be fully described using conventional methods. When panelists could associate a word with a particular position or grouping, they generated a free-form sensory profile for that fraction. After collecting and refining these descriptors, the vocabulary was standardized and then presented to the panelists to score the intensity of each fraction for each descriptor. This tasting protocol generated a coordinate matrix (x, y positions for each sample) along with an intensity scores matrix for the descriptors, which is compatible with further multivariate data analysis (Husson and Pagès, 2006) (an example of the process and its statistical treatment is provided in Appendix E of Supplementary data). At the end of the session, a second glass of the same Chasselas wine was provided to assess any taste-modulating effects of the fractions (a summary of these activities is available in Appendix F of Supplementary data). Across all experiments, over 200 distinct descriptors were generated and subsequently curated into 14 categorical groups. These descriptors, along with their English-translated categories, are illustrated in Fig. 1.

The categories illustrated in Fig. 1 were then used to consolidate the various descriptors for each group of fractions, thereby enhancing statistical power. The results of this consolidation, particularly addressing missing values for the bitter taste, are detailed in Appendix G of Supplementary data. By summing the intensity scores provided by each panelist within these harmonized categories, a comprehensive global taste profile was obtained for each sample.

### 2.3 Sensorial and chemical information gain due to fractionation

The comparison between classical tasting using free vocabulary and our *chemically informed tasting* approach is illustrated in Fig. 2.

In the classical tasting of the crude extract, only three taste attributes were detected, with bitterness accounting for over 85% of the overall taste intensity. In contrast, the *chemically informed tasting* revealed nine additional taste attributes, thereby uncovering a richer and more nuanced sensory profile. The values shown represent the sum of intensities reported by at least two panelists and were normalized by the mass of the collected fractions. Notably, although bitterness remained the dominant taste, its relative contribution was reduced from 86% in the crude extract to 46% after fractionation (see Appendix A of Supplementary data). Even seemingly minor taste notes, when consistently reported, underscore their importance in the overall sensory impression.

On the chemical side, an *automated composition assessment* was performed on both the crude extract and its fractions (Rutz and Wolfender, 2023). Fractionation increased the number of mass spectrometric features from 3,471 to 8,104. This increase not only expanded the chemical space but also improved the confidence in unique compound annotations, which rose from 1,562 to 2,327. Among these 2,327 confident annotations, 172 structures had been previously reported in Gentianaceae, including 63 compounds that were not annotated in the crude extract. These low-abundance compounds, undetected in the crude extract, may nonetheless contribute significantly to the extract’s overall taste profile.

**Fig. 3:**
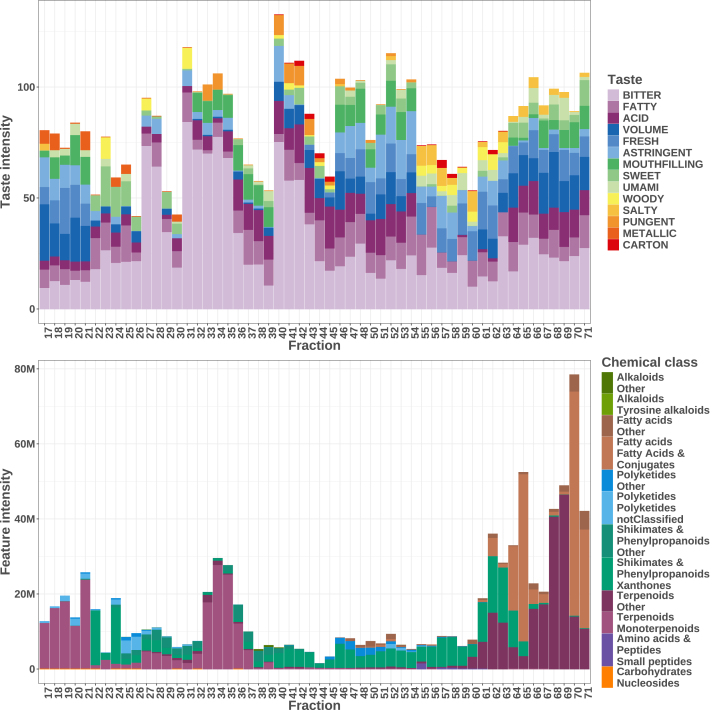
**Comparison of the *automated composition assessment* with the *chemically informed tasting* of the fractions.** The upper panel displays the assigned taste intensities for each fraction, while the lower panel presents the corresponding chemical classes identified through automated composition assessment. In the sensory panel, variations attributable to different tasting sessions are evident, underscoring the dynamic nature of human perception. Conversely, the chemical panel reveals the major contribution of terpenoids to the overall chemical profile.

Fig. 3 compares the *automated composition assessment* with the *chemically informed tasting* across the fractions. The top panel displays the attributed taste intensities per fraction, while the bottom panel shows the corresponding chemical classes. All fractions exhibited both bitter and fatty taste categories, with bitterness peaking notably in fractions 27–29, 31–36, and 40–43. Fractions 46–54, however, demonstrated a reduced perception of bitterness, coinciding with an absence of monoterpenoids and the emergence of other gustatory and trigeminal attributes such as fresh, astringent, and sweet notes. This chromatographic region is primarily correlated with the presence of xanthones and their dimers, which are characteristic of the Gentianaceae family. In the more lipophilic fractions (64–71), where triterpenes and fatty acids predominated, no distinct taste trend was recorded, suggesting that these compounds may exert minimal or inconsistent influence on taste perception. Interestingly, in the most polar fractions (17–26), despite the high abundance of monoterpenoids (especially in fractions 17–21), a significant bitter taste was not perceived.

### 2.4 Sensorial and chemical correlations

**Fig. 4:**
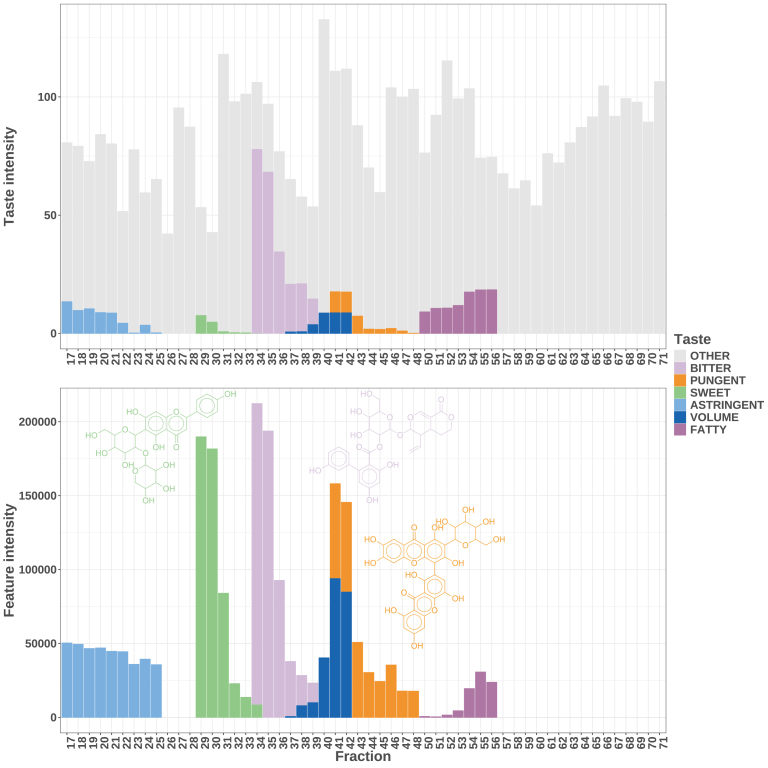
**Selected Significant Feature-Taste Intensities Correlations.** Our approach enabled us to robustly correlate specific taste attributes with distinct chemical features. For example, the bitterness in fractions 34–39 strongly correlated with the presence of Amarogentin, the pungency in fractions 41–48 with 3-*O*-Demethylswertipunicoside, and the sweetness in fractions 29–34 with Isovitexin2”-*O*-arabinoside. Additional correlations were observed for astringency, volume, and fatness, although these lacked confident molecular annotations. Notably, multiple taste attributes were linked within the same fractions (e.g., fraction 34 exhibited both bitter and sweet notes; fractions 37–39 were associated with bitter and volume attributes; fractions 41–42 correlated with both volume and pungency), and consistent patterns emerged across different tasting sessions (see Appendix B of Supplementary data). For clarity in visualization, the intensities of the bitter and pungent ions were scaled down by factors of 300 and 100, respectively.

To refine the trends observed in Fig. 3, over six million correlations between feature-taste intensity pairs were calculated. From these, 3,272 correlations (0.05%) were retained as statistically significant (p-value < 0.05 and correlation coefficient > 0.95). Fig. 4 illustrates several of the strongest positive correlations across different taste descriptors.

For example, the bitterness in fractions 34–39 showed a robust correlation with Amarogentin, while the pungency in fractions 41–48 correlated with 3-*O*-Demethylswertipunicoside. Similarly, the sweetness observed in fractions 29–34 was positively correlated with an ion annotated as Isovitexin2”-*O*-arabinoside, a compound whose sweet character could be supported by the known sweetness of its arabinose moiety (Rojas et al., 2022). Additional significant correlations for astringency, volume, and fatness are depicted in Fig. 4 in light blue, dark blue, and dark violet, respectively. Notably, some fractions exhibited correlations for multiple taste attributes simultaneously (e.g., fraction 34 was associated with both bitter and sweet notes; fractions 37–39 with bitter and volume; and fractions 41–42 with volume and pungency), with these patterns consistently observed across different tasting sessions (see Appendix B of Supplementary data). Because they were known to contribute to the bitterness of Gentianaceae spp. Wolfender et al. (1993), all ions confidently annotated as iridoids revealed that the overall bitterness of the extract correlated well with Amarogentin and Amaroswerin—two well-known bitter iridoids in *S. chirayita*—as well as with a Decentapicrin derivative. Although no prior reports have documented the bitterness or occurrence of Decentapicrin derivatives in *Swertia* spp., Decentapicrin A was previously identified alongside Amarogentin and Amaroswerin in *Gentianella nitida* (Kawahara et al., 2001). It is important to note that the correlations highlighted in Fig. 4 represent only a small subset of the overall dataset. Nevertheless, these selected correlations effectively illustrate both the strengths and limitations of our approach. For instance, molecules with similar taste profiles that elute closely may be difficult to distinguish, potentially leading to underestimation of correlations, as observed for Amaroswerin in Fig. S7. Similarly, in fractions where multiple taste-active molecules are co-eluting, mixed sensory signals can complicate correlation, yet our methodology successfully prioritized compounds with distinct taste attributes. Moreover, the retrieval of meaningful correlations across different tasting sessions underscores the robustness of our approach, even in the presence of expected inter- and intra-session variability in free vocabulary experiments.

## 3 Conclusion

We employed a combined analytical and sensory untargeted approach to identify the taste-active compounds in an ethanolic extract of *S. chirayita*. Fractionation of the extract enabled a detailed exploration of its chemical composition via automated compositional assessment. The integration of high-resolution mass spectrometry with chemically informed tasting allowed us to effectively disentangle the contributions of individual molecules to the overall taste profile. This holistic approach not only refines our understanding of taste complexity but also lays the groundwork for optimizing selective extraction or synthesis of flavoring ingredients. Our results indicate that multiple iridoids, particularly Amarogentin, Amaroswerin, and a Decentapicrin derivative, are key contributors to the extract’s bitter taste, while additional minor compounds further enrich its complexity. Moreover, certain fractions exhibited taste and trigeminal modulatory activity, highlighting the multifaceted nature of bitter perception in *S. chirayita*. These findings underscore the potential of *S. chirayita* as a valuable resource for the production of high-quality bitter beverages. Future work, including taste reconstitution and omission experiments (Rotzoll et al., 2006, Engel et al., 2002), is warranted to further validate these findings despite the challenges posed by the residual complexity of rich matrices (Choules et al., 2018). The methodological framework developed herein streamlines experimental efforts and may foster a virtuous cycle where scarce compounds enhance panelist training and improve sensory evaluations. While this study focused on taste, the integrative approach presented holds promise for application to other biological activities that exhibit less subjective variability.

## 4 Experimental

### 4.1 Plant material

The aerial parts of *S. chirayita* were supplied by Tradall SA (Batch 155174).

### 4.2 Extraction

The plant material was used for other studies, therefore high amounts of extract were needed.

1.1 kg of plant material was homogenized in a grinder and extracted at room temperature with 5 kg H_2_O and 6 kg EtOH for 4 days. The extract was then filtered and stored in inox for decantation for 10 days. Finally, the extract was concentrated under reduced pressure, freeze-dried and stored at −20 °C until further use. From 1.1 kg of plant material, 86.3 g of dried extract was obtained.

### 4.3 Fractionation

#### 4.3.1 Vacuum liquid chromatography

The first fractionation of the extract was undertaken by Vacuum Liquid Chromatography (VLC). 10 g of extract mixed with 20 g of C_18_ silica were loaded on a chromatographic system made of two layers of silica (50 g and 200 g) separated by sand. This system was first very gently (approximately 2 drops/sec) eluted with 3 × 500 mL 100% H_2_O and then 3 × 500 mL 100% EtOH. The aqueous and ethanolic parts were collected separately, with a ‘mix’ part corresponding to the dead volume of the system between both and the system washed with DCM. All VLC fractions were then concentrated under reduced pressure, freeze-dried and stored at −20 °C until further use. This procedure was repeated 3 times. From the initial 30 g of extract, a total of 27.4 g were recovered (91.3%). Mass of VLC_1 H_2_O was 12.4 g, VLC_2 (mix) was 4.2 g, VLC_3 (EtOH) was 10.4 g and VLC_4 (wash) 0.3 g.

#### 4.3.2 Medium-pressure liquid chromatography

10 g of VLC_3 were mixed with 20 g of Zeoprep® C_18_ (40–63 μm, BGB) and filled in an aluminum cartridge. Fractionation was performed using a Buchi 681 pump equipped with a Knauer UV detector and a 460 × 70 mm i.d. column loaded with Zeoprep® C_18_ as stationary phase (15–25 μm, BGB). Fractionation was performed at a flow rate of 20 mL/min using a solvent mixture of 0.1% formic acid in water (eluent A) and 0.1% formic acid in methanol (eluent B) and the following gradient: 0 min, 5% B; held for 37 min at 5% B; increased within 600 min to 100% B; washed at 100% B. The column effluent was fractionated each 250 mL into 82 sub-fractions (M01–M82). The sub-fractions were then concentrated under reduced pressure, freeze-dried and stored at −20 °C until further use. From the initial 10 g of VLC_3, a total of 7.8 g were recovered (77.8%). More details are available in Appendix A of Supplementary data.

### 4.4 Sensory analysis

This study did not involve medical applications and, therefore, was not subject to the Code of Ethics of the World Medical Association in Switzerland. However, the research project was conducted with utmost respect for the dignity, privacy, and well-being of the participants. Anonymity was maintained during data analysis and publication of the results. The participants were experienced sensory testers who voluntarily participated without any coercion. Informed consent was obtained, and the study’s objectives were clearly communicated prior to the testing sessions. Participants retained the freedom to choose whether or not to take part in the sessions.

#### 4.4.1 Panel

The tasting panel included 11 female and 5 male participants. The panelists were trained in sensory evaluation once a week for 2 to 12 years, depending on the participant, before participating in this study. These panelists were primarily trained on wine but they were used to tasting other products. Sensory sessions were run for 10 weeks (once a week) and the number of panelists varied from 9 to 12 per session. Specific training was done for this study.

#### 4.4.2 Organization

The distribution of the samples was done according to proton NMR clustering of the samples. 7 groups were formed by hierarchical clustering to get a reasonable number of samples per session and number of sessions. A cluster was tasted per week. Additionally to the samples of the clusters, panelists were asked to blindly evaluate a Chasselas wine before and after tasting, in order to assess the taste modulating properties of the cluster.

#### 4.4.3 Sample preparation

To minimize saturation and own ethanol tasting properties, all samples were dissolved in demineralized water. To avoid solubility issues, all samples were first prepared in a 60% EtOH stock solution at 10 mg/mL and then diluted in water.

#### 4.4.4 Working concentration

To evaluate appropriate working concentration, a first session was dedicated to VLC_3 tasting (ethanolic fraction). After tasting, the working concentration for all experiments was established at 3 mg/L, multiplied by the mass contribution of the Medium Pressure Liquid Chromatography (MPLC) fraction to the VLC_3 fraction. For more details, see Appendix I of Supplementary data. For cluster 3, working concentration was divided by 50 as preliminary chemical analyses revealed that they contained extremely bitter compounds. For cluster 7, working concentration was multiplied by 2, as preliminary chemical analyses revealed that it represented the largest amount of the extract’s mass.

### 4.5 Data acquisition

#### 4.5.1 Mass spectrometry

Chromatographic separation was performed on a Waters Acquity UHPLC system interfaced to a Corona™ Veo™ RS Charged Aerosol Detector (CAD) and a Q-Exactive Focus mass spectrometer, using a heated electrospray ionization (HESI-II) source. Thermo Scientific Xcalibur 3.1 software was used for instrument control. The conditions were as follows: column, Waters BEH C_18_ 150 × 2.1 mm, 1.7 μm; mobile phase, (A) water with 0.1% formic acid; (B) acetonitrile with 0.1% formic acid; flow rate, 400 μl⋅min^-1^; injection volume, 6 μl; gradient, isocratic at 5% B for 0.5 min linear gradient of 5%–100% B over 28 min and isocratic at 100% B for 12 min. The optimized HESI-II parameters were as follows: source voltage, 3.5 kV (pos); sheath gas flow rate N_2_, 55 units; auxiliary gas flow rate, 15 units; spare gas flow rate, 3.0; capillary temperature, 350.00 °C, S-Lens RF Level, 45. The mass analyzer was calibrated using a mixture of caffeine, methionine–arginine–phenylalanine–alanine–acetate (MRFA), sodium dodecyl sulfate, sodium taurocholate, and Ultramark 1621 in an acetonitrile/methanol/water solution containing 1% formic acid by direct injection. The data-dependent MS/MS events were performed on the three most intense ions detected in full scan MS (Top3 experiment). The MS/MS isolation window width was 1 Da, and the stepped normalized collision energy (NCE) was set to 15, 30 and 45 units. In data-dependent MS/MS experiments, full scans were acquired at a resolution of 35,000 FWHM (at *m/z* 200) and MS/MS scans at 17,500 FWHM both with an automatically determined maximum injection time. After being acquired in a MS/MS scan, parent ions were placed in a dynamic exclusion list for 2.0 s. A custom exclusion list was used. An Acquity UHPLC PDA detector was used to acquire UV spectra which were detected from 200 to 500 nm. An analytical split was used with a split ratio of 9:1 (CAD:MS). CAD parameters were: evaporation temperature at 40 °C, 5 bar N_2_, power function 1. The acquisition parameters were the same as described previously (Rutz and Wolfender, 2023). Analyses were carried in positive mode only.

#### 4.5.2 Nuclear magnetic resonance spectroscopy

NMR experiments (^1^H, ^13^C, and 2D) were performed using a Bruker® Avance III HD 600 (14,1 T) instrument (Bruker BioSpin GmbH, Rheinstetten,Germany) with trimethylsilane (TMS) as internal standard. The details of the sequences are available in the respective subfolders archived on Zenodo (https://zenodo.org/records/14414272) (Rutz et al., 2024).

### 4.6 Data conversion

#### 4.6.1 Mass spectrometry

All raw data files were converted to .mzML open format Martens et al. (2011) using ThermoRawFileParser v.1.4.5 (Hulstaert et al., 2019). The generic command used was:

mono ThermoRawFileParser.exe -d $DIRECTORY --allDetectors --format=2

### 4.7 Data processing

#### 4.7.1 Mass spectrometry

##### 4.7.1.1 Features’ extraction

MS features were extracted and informed using mzmine (4.4.0) (Pluskal et al., 2010, Heuckeroth et al., 2024). For the enriched extract, 3 replicates were measured, while fractions were measured once. First, mass detection was performed with a minimal intensity of 1.0E^4^ for MS^1^ and 0 for MS^2^. Chromatograms were built with minimum 4 consecutive scans above 1.0E^4^ and a minimal absolute height of 5.0E^4^. The *m/z* tolerance was set to 12.0 ppm. Chromatograms were then smoothed using Savitzky Golay algorithm and a window of 5. Features were then resolved using the local minimum feature resolver with a chromatographic threshold of 90%, a minimum absolute height of 1.0E^5^, a minimal ratio of peak top over edge of 1.80, a peak duration range from 0.01 to 1.50 min and minimum 5 data points. ^13^C isotope filter was then applied using an intra sample tolerance of 6.0 ppm and 0.04 min. Monotonic shape was required, with a maximum charge of 2. Further, isotopes were searched using again a 6.0 ppm tolerance and a maximum charge of 2. Features lists were then aligned using a 12.0 ppm tolerance with a weight of 3 times the one of 0.15 min. Only features with a valid isotopic pattern and at least one associated fragmentation spectrum were retained. For both the 3 extract replicates and the fractions, features had to be present in at least 3 samples. Gap filling was then performed using an intensity tolerance of 20%, 12.0 ppm and 0.15 min. The list was further refined, removing duplicates using a tolerance of 6.0 ppm and 0.15 min. For fractions, features present in all samples were removed. Features were then correlated using a 0.08 min tolerance, an intensity threshold of 1.0E^5^, a minimum of 5 data points, of which 2 on edge. Correlation measure was PEARSON on both shape and height with a minimal correlation of 65%. Ion identity networking (Schmid et al., 2021) was performed using a maximum charge of 2, a maximum molecules per cluster of 2, and the following adducts list: [M+H]^＋^,[M+NH_4_]^＋^,[M+Na]^＋^,[M-H+2Na]^＋^,[M+2H]^2+^,[M+H+NH_4_]^2+^,[M+H+Na]^2+^, together with the following modifications list: [M-C_6_H_10_O_5_],[M-C_6_H_10_O_4_], [M-2H_2_O],[M-H_2_O], [M-NH_3_], [M-CH_3_], [M-C_2_H_3_N]. Finally, ion identities were added using a two step approach, first with an extended range of adducts, then with an extended range of modifications (see batch file). All parameters were given in the form of a batch file, archived within the project’s main repository as described later.

##### 4.7.1.2 Features’ annotation

Features were annotated using a multi-tool approach already described in Rutz et al., 2019, Rutz and Wolfender, 2023.

###### 4.7.1.2.1 Sirius

Sirius annotations were performed in batch mode using Sirius 6.0.7 (Dührkop et al., 2019). Compounds above 800 *m/z* were omitted annotation was performed using Orbitrap default parameters. Adducts were the ones already defined in the previous subsection. Formulas were annotated using bottom-up approach, with default parameters (Xing et al., 2023). Formulas were annotated using ZODIAC, with default parameters (Ludwig et al., 2020). CSI:FingerID fingerprints were computed using default parameters (Dührkop et al., 2015). Compound classes were annotated using CANOPUS using default parameters (Dührkop et al., 2020). Confidence scores were calculated through COSMIC (Hoffmann et al., 2021). De novo structure annotation was also performed using MSNovelist (Stravs et al., 2022).

###### 4.7.1.2.2 In silico library

The in silico library used was generated as described in Allard et al. (2023). The SMILES used were coming from Rutz et al. (2023). CFM 4.0 was used for in silico fragmentation (Wang et al., 2021). Parameters are available at https://github.com/mandelbrot-project/spectral_lib_builder.

###### 4.7.1.2.3 Taxonomically informed metabolite annotation

Taxonomically Informed Metabolite Annotation was performed using all the above mentioned inputs. It was performed using TIMA (2.11.0) with default parameters, and a maximum number of candidates of 500 (Rutz et al., 2019, Rutz, 2024b). The resulting parameters files were archived within the project’s main repository as described later.

##### 4.7.1.3 Features’ correlation

Because of its sensitivity, and because minor compounds might also have a very strong impact on taste, MS was chosen over NMR or CAD to calculate correlations to the reported taste intensities.

###### 4.7.1.3.1 Correlation to charged aerosol detector

The extracted MS features were used to extract their related MS peak shapes and compare them to the CAD peaks detected in the same retention time window, as described in Rutz and Wolfender (2023). Default parameters from cascade (v.0.0.0.9000) were used. Peaks were detected using routine functions imported from chromatographR package (Bass, 2023). Peak shapes were then compared using the compareChromatograms function from the MSnbase package with the closest method as argument (Gatto et al., 2020).

###### 4.7.1.3.2 Correlation to taste

Only tastes reported at least by two panelists were kept, and their intensities summed. Only ions detected in at least 5 fractions were kept. Missing values were imputed using half of the lowest value. Then, MS intensities and taste intensities were correlated using the following steps: First, segments of fractions including 5 to 9 fractions were generated. For each one of these segments, the distribution of taste and ion intensities were evaluated using a Shapiro–Wilk test (Royston, 1982). If they significantly varied from a normal distribution (p-value < 0.05), correlation was computed using Kendall’s τ, else it was computed using Pearson’s coefficient (Hollander et al., 2015). P-values were then adjusted using Benjamini–Hochberg method (Benjamini and Hochberg, 1995). The correlations were refined to include only those where the evaluated ion’s intensity ranked among the top five intensity values of the ion at least four times.

#### 4.7.2 Nuclear magnetic resonance spectroscopy

The data were processed using AlpsNMR (4.8.0) (Madrid-Gambin et al., 2020). The ppm values of samples 69, 70 and 71 were manually fixed as they deviated significantly from the rest of the experiment. Then, signals were phased automatically, using background correction and default parameters. Signals were then interpolated from 0 to 14.85 ppm using automatically calculated resolution. Peaks were then detected using lorentzian fits, signals normalized, and peaks integrated using a peak width of 0.01 ppm. Finally, samples were clustered on the square root of their peak areas, using canberra distance measure (Lance and Williams, 1966) and ward.D2 agglomeration method (Ward, 1963). 7 clusters were formed from the resulting dendrogram and used further to organize tasting sessions.

### 4.8 Code availability

All programs written for this work can be found in the following repository: https://github.com/Adafede/sapid. Version 0.0.0.9000 was archived on Zenodo (https://zenodo.org/records/14616396) (Rutz, 2025).

Main dependencies were AlpsNMR (4.8.0) (Madrid-Gambin et al., 2020), cascade (0.0.0.9000) (Rutz and Wolfender, 2023, Rutz, 2024a) (relying heavily on MSnbase (2.28.1) (Gatto et al., 2020) and Spectra (1.12.0) (Rainer et al., 2022)), dendextend (1.19.0) (Galili, 2015), FactoMineR (2.11) (Lê et al., 2008), forcats (1.0.0) (Wickham, 2023), ggbump (0.1.0) (Sjoberg, 2020), ggplot2 (3.5.1) (Wickham, 2016), ggpubr (0.6.0) (Kassambara, 2023), ggraph (2.2.1) (Pedersen, 2024), igraph (2.1.2) (Csardi and Nepusz, 2006), khroma (1.14.0) (Frerebeau, 2024), NMRphasing (1.0.5) (Jiang, 2024), purrr (1.0.2) (Vaughan and Dancho, 2022), readxl (1.4.3) (Wickham and Bryan, 2023), scales (1.3.0) (Wickham et al., 2023), SensoMineR (1.27) (Husson et al., 2023), stringi (1.8.4) (Gagolewski, 2022), tibble (3.2.1) (Müller and Wickham, 2023), tidytable (0.11.1) (Fairbanks, 2024), and treemapify (2.5.6) (Wilkins, 2023).

## CRediT authorship contribution statement

**Adriano Rutz:** Conceptualization, Data curation, Formal analysis, Investigation, Methodology, Project administration, Software, Supervision, Validation, Visualization, Writing – original draft, Writing – review and editing. **Pascale Deneulin:** Methodology, Resources, Supervision, Writing – review and editing. **Ivano Tonutti:** Funding acquisition, Resources. **Benoît Bach:** Resources, Writing – review and editing. **Jean-Luc Wolfender:** Funding acquisition, Resources, Supervision, Writing – review and editing.

## Declaration of competing interest

The authors declare the following financial interests/personal relationships which may be considered as potential competing interests: Jean-Luc Wolfender reports financial support was provided by Tradall SA. Author previously employed by TRADALL S.A.: A.R. If there are other authors, they declare that they have no known competing financial interests or personal relationships that could have appeared to influence the work reported in this paper.

## Data Availability

The sensorial data were converted to open format and archived on Zenodo( https://zenodo.org/records/14616396) (Rutz, 2025). The raw NMR data were archived on Zenodo ( https://zenodo.org/records/14414272) (Rutz et al., 2024), and on nmrXiv ( https://doi.org/10.57992/nmrxiv.p90) (Rutz et al., 2025), as recommended by McAlpine et al. (2019). The raw UHPLC-MS/MS data (coupled to PDA and CAD) were converted to open format and archived on MassIVE ( https://massive.ucsd.edu/ProteoSAFe/dataset.jsp?accession=MSV000096654), together with ReDU compliant metadata (Jarmusch et al., 2020).

## Appendices Supplementary data

MMC S1 Supplementary figures.

MMC S2 Supplementary table. Masses of the MPLC fractions.
