# Supplementary material for: SAPID: A Strategy to Analyze Plant Extracts Taste In Depth. Application to the complex taste of Swertia chirayita (Roxb.) H.Karst
Source: Curr Res Food Sci. 2025 Apr 5;10:101043. doi: 10.1016/j.crfs.2025.101043 (PMC12051061; doi:10.1016/j.crfs.2025.101043)
Supplement: MMC S1 — Supplementary figures. [file mmc1.pdf]

//dx.doi.org/10.1093/chromsci/bmac018, doi:10.1093/chromsci/bmac018.

Shukla, S., Bafna, K., Sundar, D., Thorat, S.S., 2014. The bitter barricading of prostaglandin biosynthesis pathway: Understanding the molecular mechanism of selective cyclooxygenase-2 inhibition by amarogentin, a secoiridoid glycoside from swertia chirayita. PLoS ONE 9, e90637. URL: http://dx.doi.org/10.1371/journal.pone.0090637, doi:10.1371/journal.pone.0090637.

Sjoberg, D., 2020. ggbump: Bump Chart and Sigmoid Curves. URL: https://CRAN.R-project.org/package=ggbump. r package version 0.1.0.

Stravs, M.A., Dührkop, K., Böcker, S., Zamboni, N., 2022. Msnovelist: de novo structure generation from mass spectra. Nature Methods 19, 865–870. URL: http://dx.doi.org/10.1038/s41592-022-01486-3, doi:10.1038/s41592-022-01486-3.

Suryawanshi, S., Mehrotra, N., Asthana, R.K., Gupta, R.C., 2006. Liquid chromatography/tandem mass spectrometric study and analysis of xanthone and secoiridoid glycoside composition of swertia chirata, a potent antidiabetic. Rapid Communications in Mass Spectrometry 20, 3761–3768. URL: http://dx.doi.org/10.1002/rcm.2795, doi:10.1002/rcm.2795.

Vaughan, D., Dancho, M., 2022. furrr: Apply Mapping Functions in Parallel using Futures. URL: https://CRAN.R-project.org/package=furrr. r package version 0.3.1.

Wang, F., Liigand, J., Tian, S., Arndt, D., Greiner, R., Wishart, D.S., 2021. Cfm-id 4.0: More accurate esi-ms/ms spectral prediction and compound identification. Analytical Chemistry 93, 11692–11700. URL: http://dx.doi.org/10.1021/acs.analchem.1c01465, doi:10.1021/acs.analchem.1c01465.

Ward, J.H., 1963. Hierarchical grouping to optimize an objective function. Journal of the American Statistical Association 58, 236–244. URL: http://dx.doi.org/10.1080/01621459.1963.10500845, doi:10.1080/01621459.1963.10500845.

Wickham, H., 2016. ggplot2: Elegant Graphics for Data Analysis. Springer-Verlag New York. URL: https://ggplot2.tidyverse.org.

Wickham, H., 2023. forcats: Tools for Working with Categorical Variables (Factors). URL: https://CRAN.R-project.org/package=forcats. r package version 1.0.0.

Wickham, H., Bryan, J., 2023. readxl: Read Excel Files. URL: https://CRAN.R-project.org/package=readxl. r package version 1.4.3.

Wickham, H., Pedersen, T.L., Seidel, D., 2023. scales: Scale Functions for Visualization. URL: https://CRAN.R-project.org/package=scales. r package version 1.3.0.

Wilkins, D., 2023. treemapify: Draw Treemaps in 'ggplot2'. URL: https://CRAN.R-project.org/package=treemapify. r package version 2.5.6.

Wolfender, J.L., Hamburger, M., Hostettmann, K., Msonthi, J.D., Mavi, S., 1993. Search for bitter principles in chironia species by lc-ms and isolation of a new secoiridoid diglycoside from chironia krebsii. Journal of Natural Products 56, 682–689. URL: http://dx.doi.org/10.1021/np50095a004, doi:10.1021/np50095a004.

Wölfle, U., Schempp, C.M., 2018. Bitterstoffe–von der traditionellen verwendung bis zum einsatz an der haut. Zeitschrift für Phytotherapie 39, 210–215. URL: http://dx.doi.org/10.1055/a-0654-1711, doi:10.1055/a-0654-1711.

Xing, S., Shen, S., Xu, B., Li, X., Huan, T., 2023. Buddy: molecular formula discovery via bottom-up ms/ms interrogation. Nature Methods 20, 881–890. URL: http://dx.doi.org/10.1038/s41592-023-01850-x, doi:10.1038/s41592-023-01850-x.

Yan, J., Tong, H., 2022. An overview of bitter compounds in foodstuffs: Classifications, evaluation methods for sensory contribution, separation and identification techniques, and mechanism of bitter taste transduction. Comprehensive Reviews in Food Science and Food Safety 22, 187–232. URL: http://dx.doi.org/10.1111/1541-4337.13067, doi:10.1111/1541-4337.13067.

Zhao, A., Jeffery, E.H., Miller, M.J., 2022. Is bitterness only a taste? the expanding area of health benefits of brassica vegetables and potential for bitter taste receptors to support health benefits. Nutrients 14, 1434. URL: http://dx.doi.org/10.3390/nu14071434, doi:10.3390/nu14071434.

6. Appendices

A. Masses of the MPLC fractions

B. ^{1}H NMR of the MPLC fractions

C. Chromatogram of the enriched extract compared to the chromatograms of the MPLC fractions

D. Variation of the scoring of Chasselas among all experiments

E. Initial analysis of the sensory results

F. Summary of the taste modulating activity for each group of fractions

G. Matrices of fractions reported as bitter before and after vocabulary curation

H. Correlations of the intensities of features confidently annotated as iridoids and bitter taste

I. Determination of the concentration used for tasting

Table S1
Masses of the MPLC fractions.

Fraction	Mass [mg]		Fraction	Mass [mg]		Fraction	Mass [mg]		Fraction	Mass [mg]
M_01	0.0		M_22	143.0		M_43	149.7		M_64	51.1
M_02	0.0		M_23	195.3		M_44	127.9		M_65	59.8
M_03	145.3		M_24	199.6		M_45	156.7		M_66	60.1
M_04	91.9		M_25	143.8		M_46	167.7		M_67	69.7
M_05	0.0		M_26	122.4		M_47	167.4		M_68	36.1
M_06	0.0		M_27	115.6		M_48	157.8		M_69	16.6
M_07	0.0		M_28	116.2		M_49	0.0		M_70	33.8
M_08	0.0		M_29	118.6		M_50	150.7		M_71	63.3
M_09	0.0		M_30	115.3		M_51	163.0		M_72	58.6
M_10	0.0		M_31	118.4		M_52	125.2		M_73	146.6
M_11	0.0		M_32	156.1		M_53	126.4		M_74	144.4
M_12	0.0		M_33	184.4		M_54	95.0		M_75	124.5
M_13	4.0		M_34	210.1		M_55	136.2		M_76	111.9
M_14	13.4		M_35	185.2		M_56	152.7		M_77	76.2
M_15	36.1		M_36	141.0		M_57	127.1		M_78	63.8
M_16	26.2		M_37	123.9		M_58	130.5		M_79	60.5
M_17	99.1		M_38	128.7		M_59	115.6		M_80	96.8
M_18	123.0		M_39	144.8		M_60	90.7		M_81	47.7
M_19	64.3		M_40	152.7		M_61	96.3		M_82	15.0
M_20	89.6		M_41	119.7		M_62	38.2			
M_21	99.1		M_42	168.4		M_63	61.7		**Total**	**7768.2**

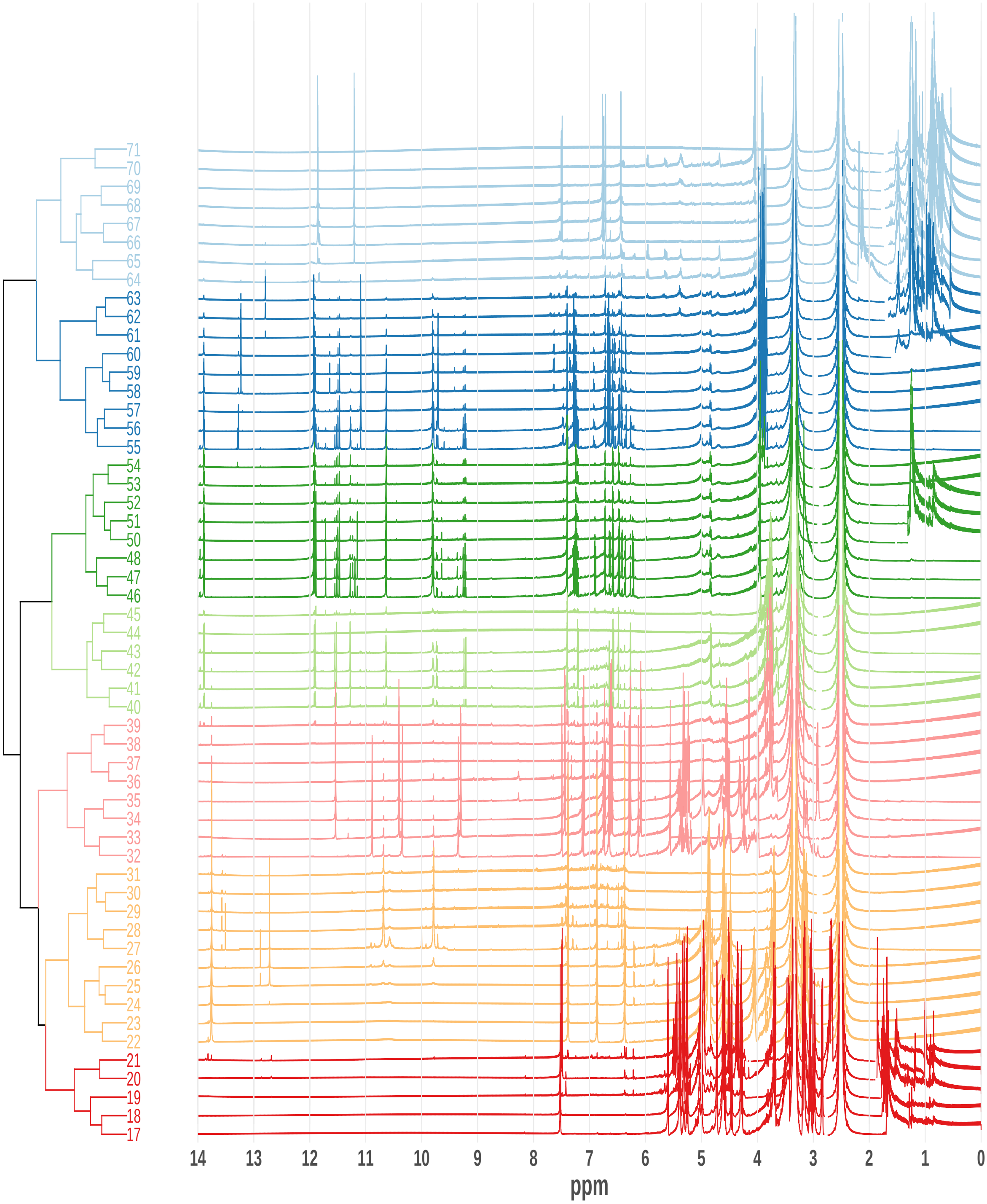

Figure S1: **^{1}H NMR of the MPLC fractions.** Fractions are colored by the group they belong to after clustering. In this case, clusters followed chromatographic order, but this must not necessarily be the case.

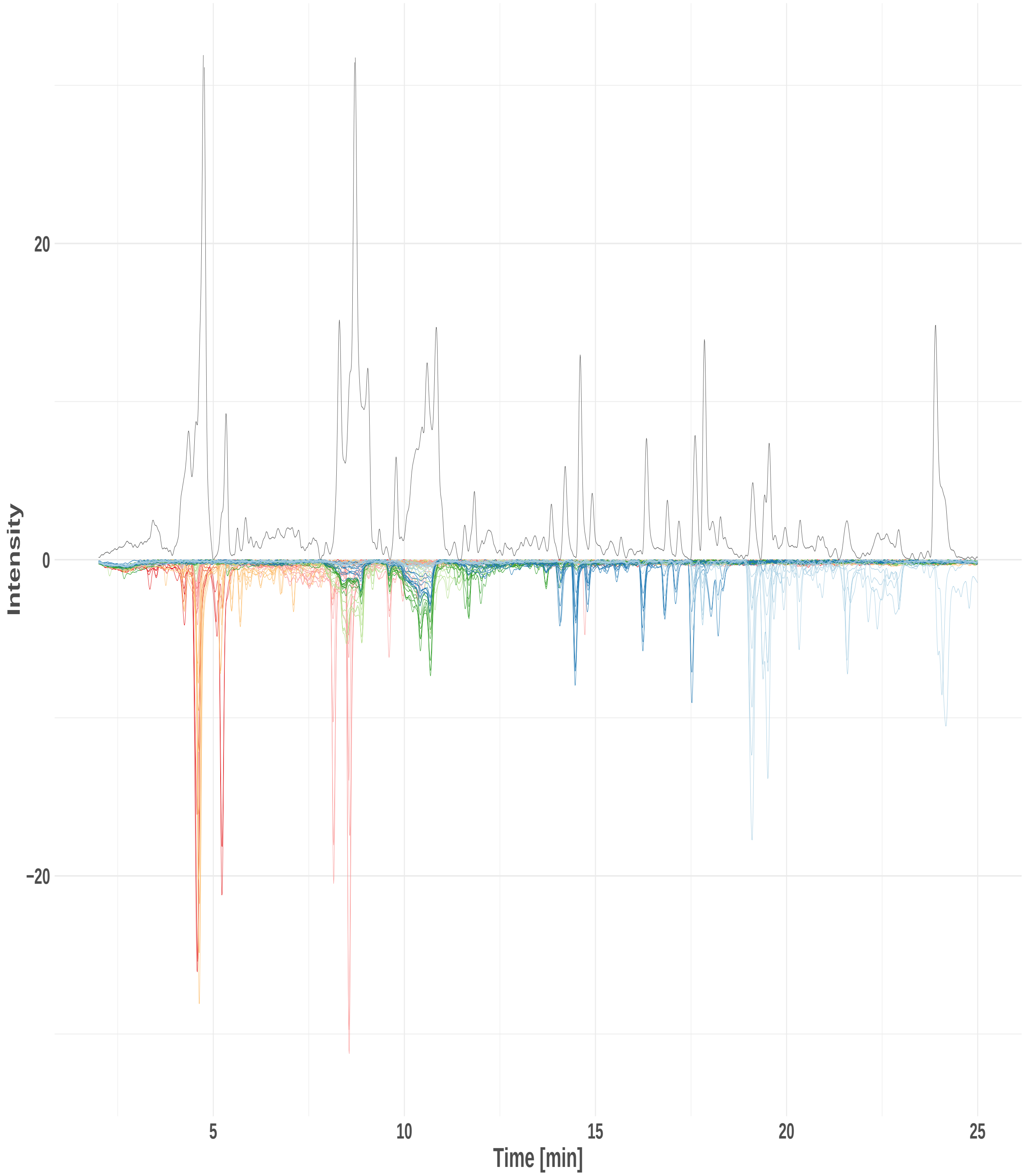

Figure S2: Chromatogram of the enriched extract compared to the chromatograms of the MPLC fractions. The chromatogram of the enriched extract is presented on the top, whereas the chromatograms of the MPLC fractions are presented on the bottom. MPLC fractions are colored according to the clustering made in Appendix B. CAD signal preprocessed as described in Rutz and Wolfender (2023). Except for the peaks at the end of the chromatogram (those fractions were discarded as being too apolar), the profiles of the enriched extract and the fractions look almost identical.

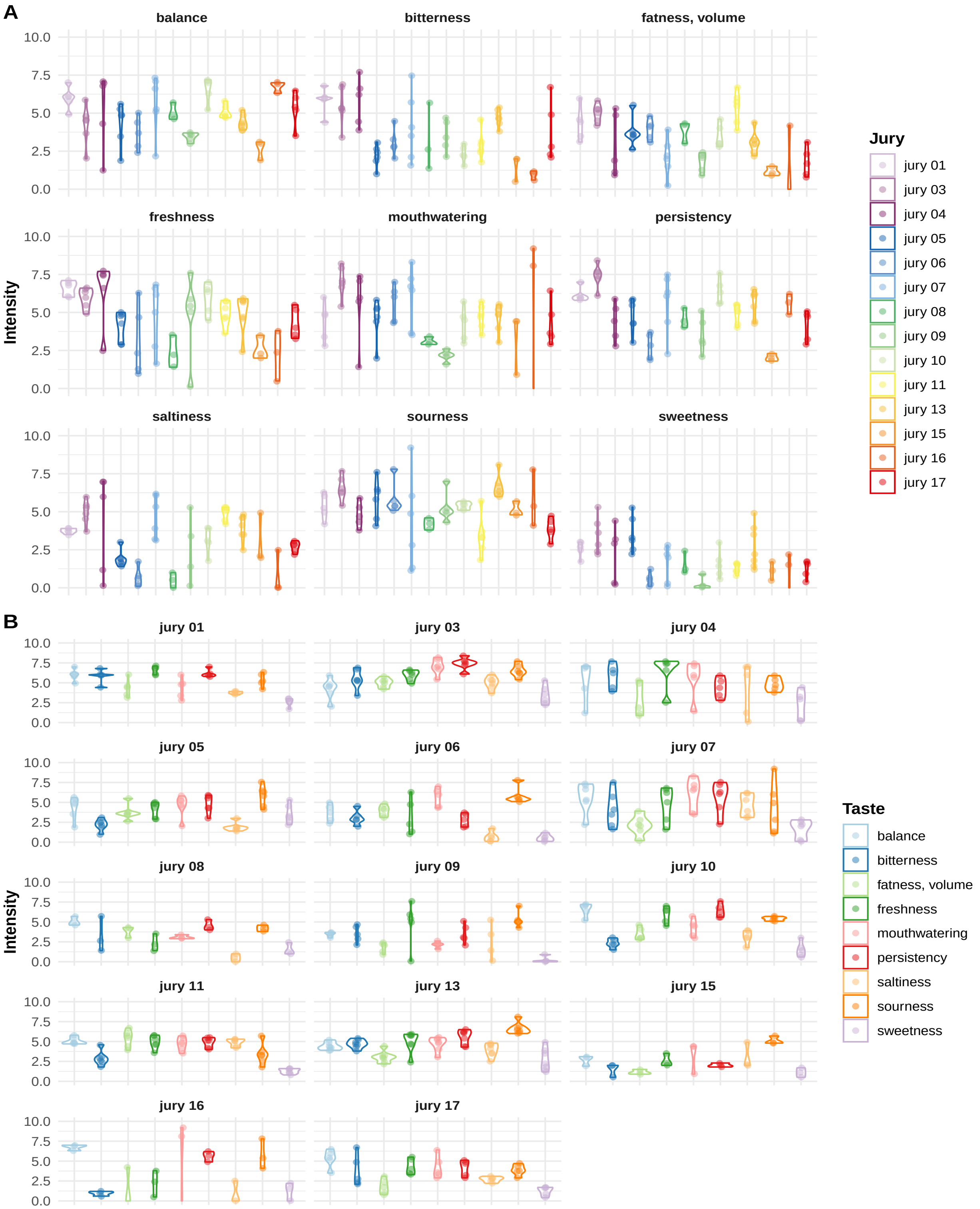

Figure S3: Variation of the scoring of Chasselas among all experiments. In panel A, variations are faceted by taste with jury as color. In panel B, variations are faceted by jury with taste as color.

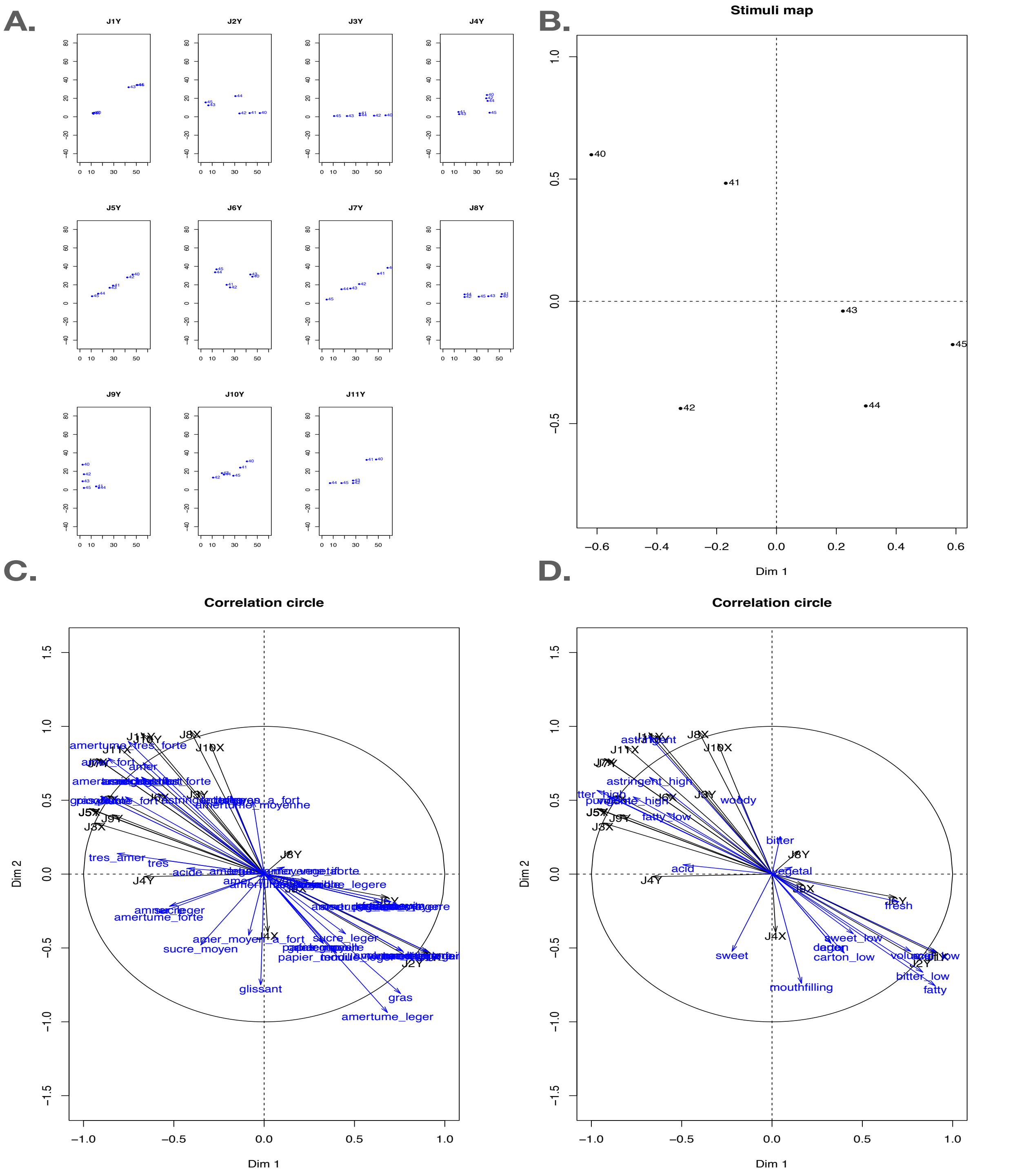

Figure S4: Initial analysis of the sensory results. The positions of each fraction for each panelist are shown in panel A. Using the INDSCAL model Husson and Pagès (2006), a summarized stimuli map is obtained (panel B). This map can then be compared to the correlation circle of the attributed descriptors (panel C). Eventually, a curation of the vocabulary used can occur, as in panel D.

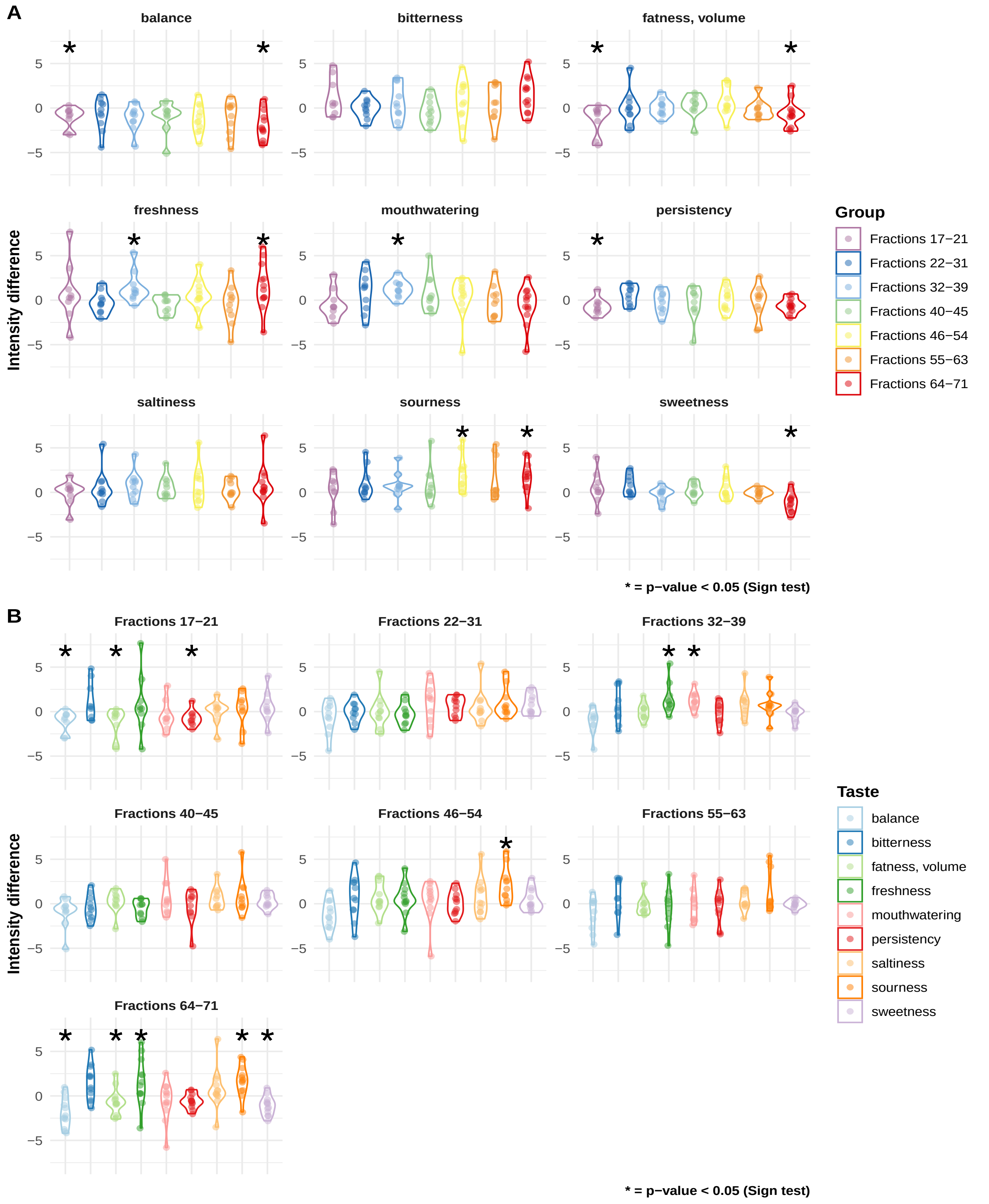

Figure S5: Summary of the taste modulating activity for each group of fractions. The values correspond to the score given to the Chasselas sample tasted after fractions tasting in comparison to the one tasted before fractions. In panel A, variations are grouped by experiment with taste as the variable. In panel B, variations are grouped by taste with experiment as the variable. Stars denote statistical significance according to Sign test (`https://en.wikipedia.org/wiki/Sign_test`).

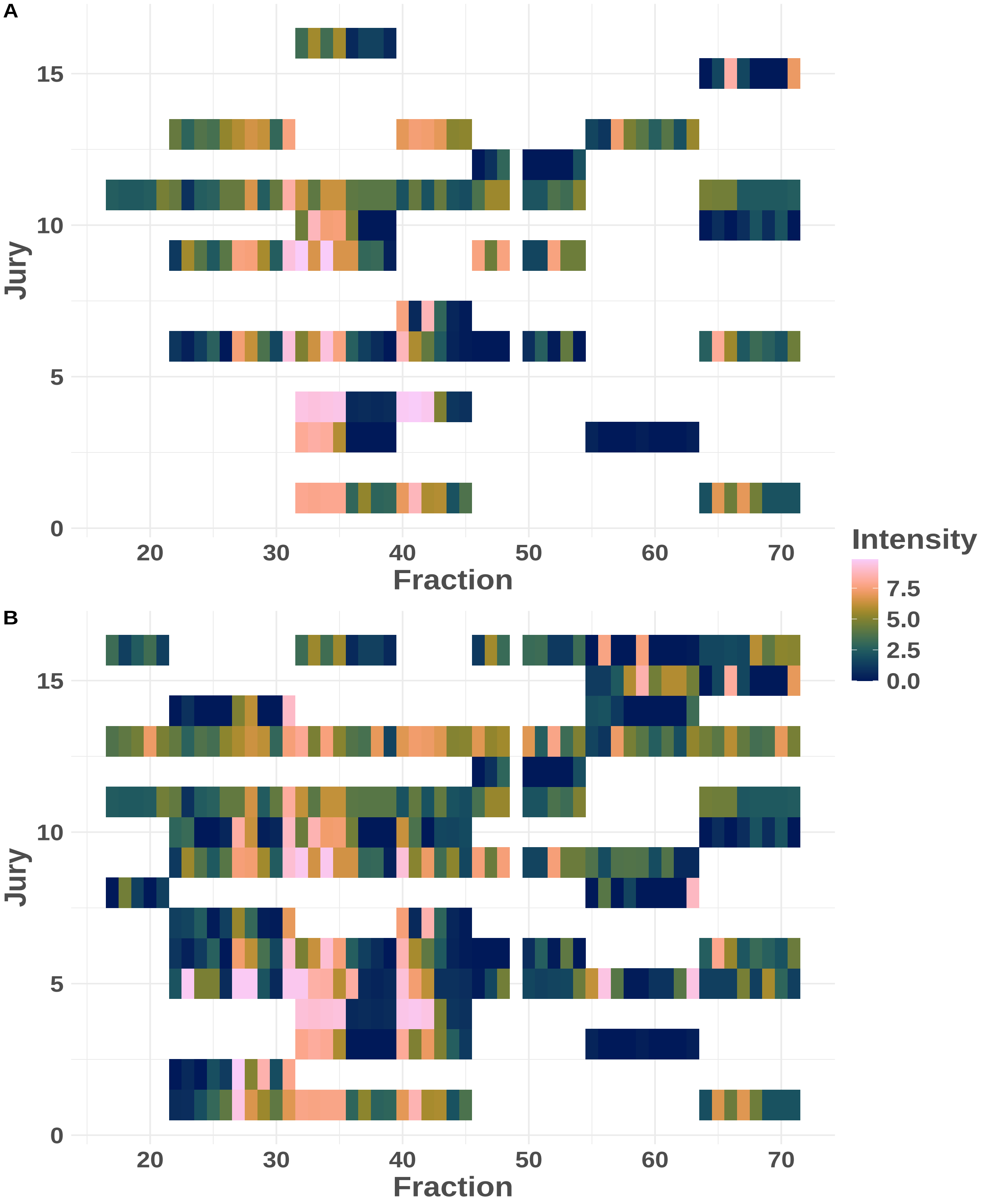

Figure S6: Matrices of fractions reported as bitter before and after vocabulary curation. In panel A, values correspond to the term 'amer' (bitter in French) only. In panel B, terms related to bitterness were grouped together as illustrated in Figure 1

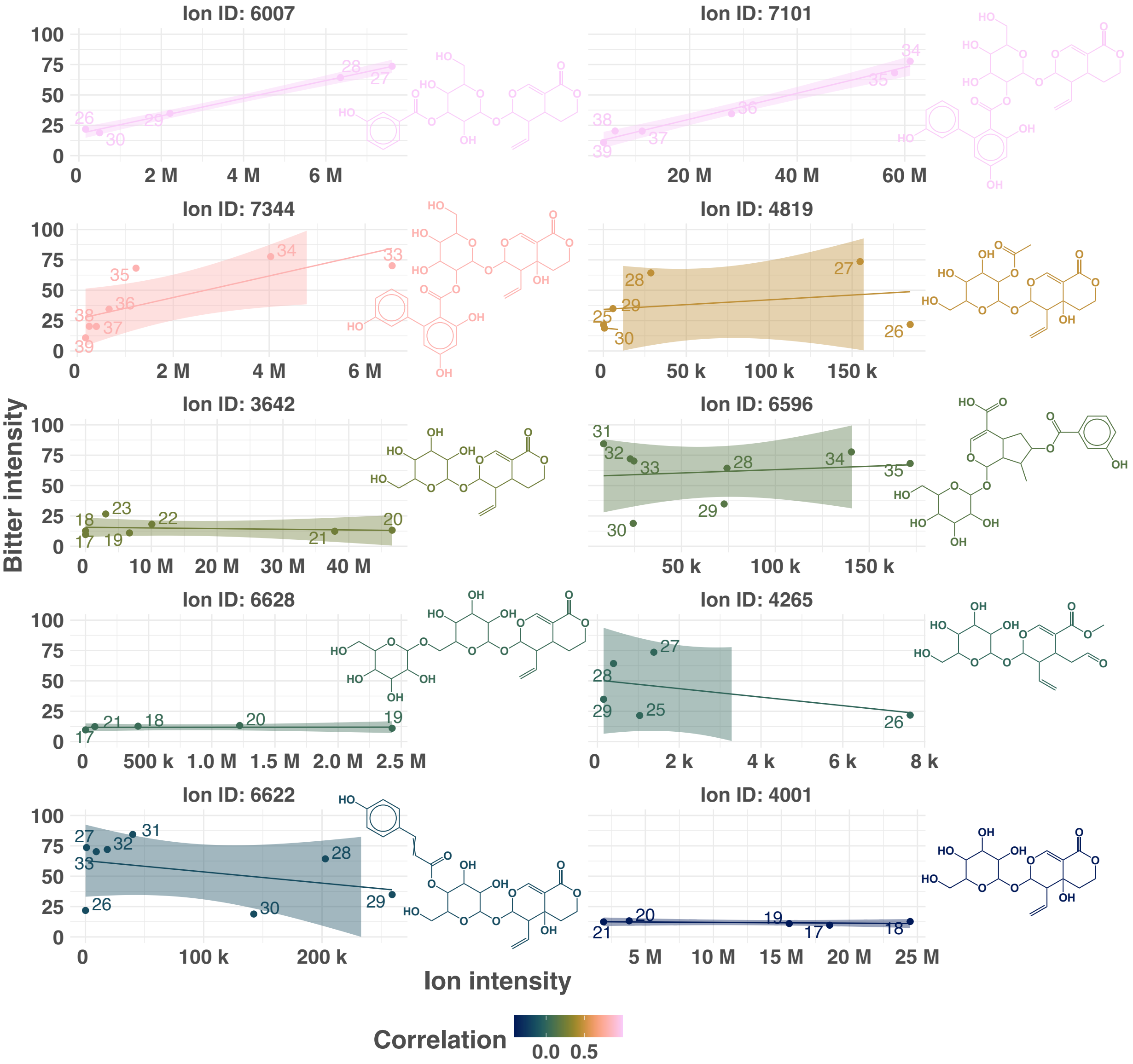

Figure S7: Correlations of the intensities of features confidently annotated as iridoids and bitter taste. The two first ions, annotated as a Decentapicrin derivative (6007) and Amarogentin (7101) respectively are well correlated to bitterness. The ion annotated as Amaroswerin (7344) has a correlation of 0.81, while other ions annotated as iridoids are not correlated, and thus probably not contributing to the bitter taste of the extract. While the presented set of 10 iridoids is not sufficient to draw any conclusions, interesting trends can be observed and larger sets could lead to interesting structure-activity relationships.

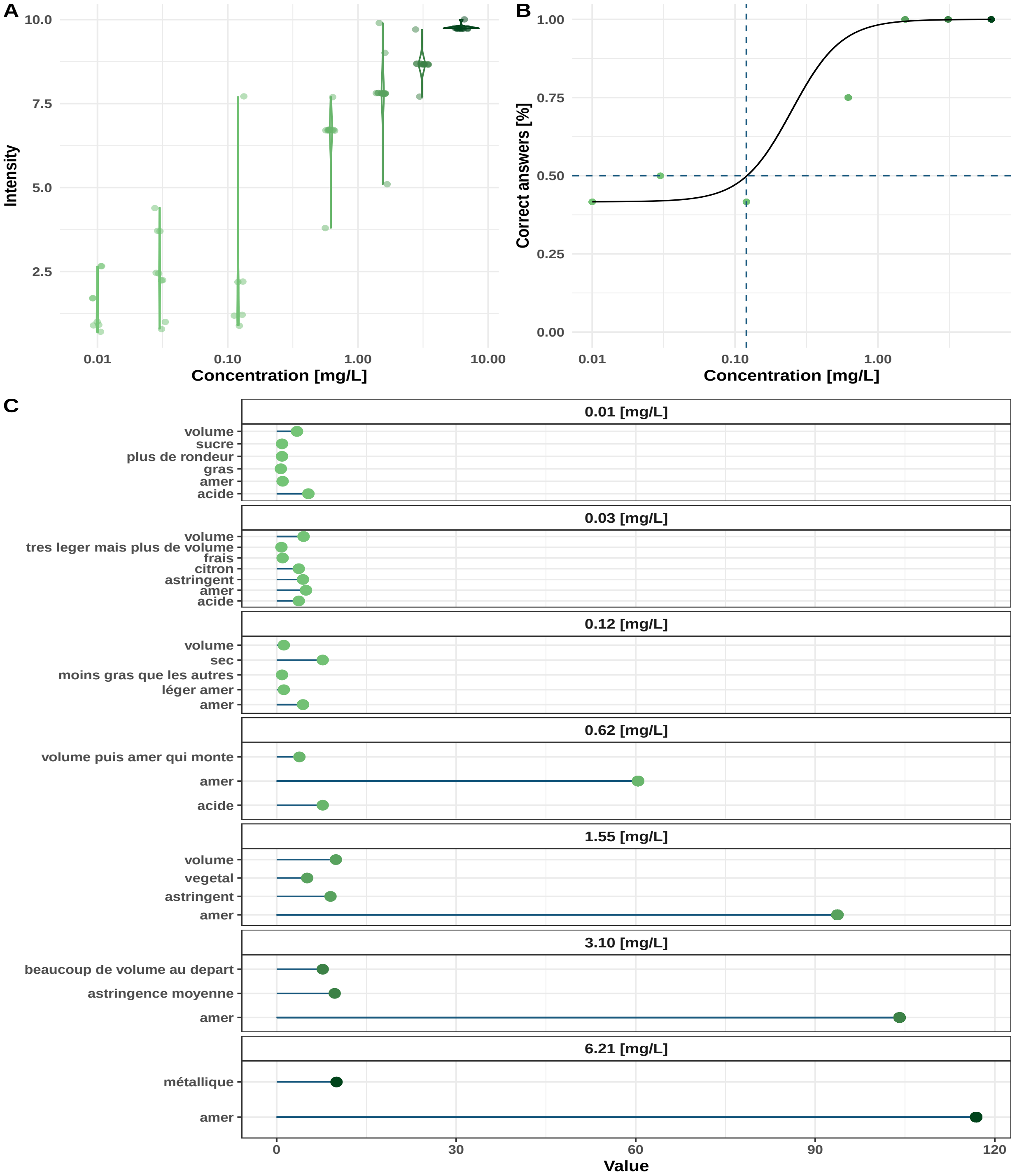

Figure S8: Determination of the concentration used for tasting. Panel A represents the intensity score given to the sample in a triangle test as a function of the enriched extract concentration (only if the answer was correct). Panel B represents the number of correct answers in a triangle test as a function of sample concentration. Finally, panel C represents the occurrence of the descriptors attributed to the sample, multiplied by the intensity given to the sample, as a function of concentration. For more information about triangle test in sensory analysis, see https://www.sensorysociety.org/knowledge/sspwiki/Pages/Triangle%20Test.aspx
